# Designing a Novel Monolayer *β*-CSe for High Performance Photovoltaic Device: An Isoelectronic Counterpart of Blue Phosphorene

**DOI:** 10.3390/nano9040598

**Published:** 2019-04-11

**Authors:** Qiang Zhang, Yajuan Feng, Xuanyu Chen, Weiwei Zhang, Lu Wu, Yuexia Wang

**Affiliations:** 1 Key Laboratory of Nuclear Physics and Ion-beam Application (MOH), Institute of Modern Physics, Fudan University, Shanghai 200433, China; 14110200002@fudan.edu.cn (Y.F.); 18210200006@fudan.edu.cn (X.C.); 17110200015@fudan.edu.cn (W.Z.); yaowang@fudan.edu.cn (Y.W.); 2 The First Sub–Institute, Nuclear Power Institute of China, Chengdu 610005, China; lulurichard.student@sina.com

**Keywords:** density functional theory, mechanical behaviors, electronic properties, type-II heterostructure, photocatalytic properties

## Abstract

Using the first-principles method, an unmanufactured structure of blue-phosphorus-like monolayer CSe (*β*-CSe) was predicted to be stable. Slightly anisotropic mechanical characteristics in *β*-CSe sheet were discovered: it can endure an ultimate stress of 5.6 N/m at 0.1 along an armchair direction, and 5.9 N/m at 0.14 along a zigzag direction. A strain-sensitive transport direction was found in *β*-CSe, since *β*-CSe, as an isoelectronic counterpart of blue phosphorene (*β*-P), also possesses a wide indirect bandgap that is sensitive to the in-plane strain, and its carrier effective mass is strain-dependent. Its indirect bandgap character is robust, except that armchair-dominant strain can drive the indirect-direct transition. We designed a heterojunction by the *β*-CSe sheet covering *α*-CSe sheet. The band alignment of the *α*-CSe/*β*-CSe interface is a type-II van der Waals *p*-*n* heterojunction. An appreciable built-in electric field across the interface, which is caused by the charges transfering from *β*-CSe slab to *α*-CSe, renders energy bands bending, and it makes photo-generated carriers spatially well-separated. Accordingly, as a metal-free photocatalyst, *α*-CSe/*β*-CSe heterojunction was endued an enhanced solar-driven redox ability for photocatalytic water splitting via lessening the electron-hole-pair recombination. This study provides a fundamental insight regarding the designing of the novel structural phase for high-performance light-emitting devices, and it bodes well for application in photocatalysis.

## 1. Introduction

Two-dimensional (2D) families spark tremendous research enthusiasm that is rooted in their exceptional and superior properties [1,2], which is governed by special structure characteristics and quantum size effects, and promising applications in cutting-edge optoelectronic and photonic devices [3,4], supercapacitors [5,6,7], lithium-ion battery [8,9,10], efficient ultra-violet photodetector [11], photocatalysis [12,13,14], field effect transistors [15,16], superior gas sensor [17], and so on. These families, which include semi-metallic graphene (G), semiconducting transition metal dichalcogenides (TMDs), black phosphorene (*α*-P), and insulating hexagonal boron nitride (*h*-BN), have revealed extensive extraordinary performances. Graphene, which is a zero bandgap semimetal, was reported to possess exceptionally high mobility of carriers and splendid mechanical strength [18,19,20]. MoS_2_, as a typical representative of TMDs, possesses an optimal optical direct band gap [21], high on/off ratios [22], and superior self-healing performances in air [23]. *α*-P, an atomic ultrathin sheet, which maintains an appropriate direct band gap, was revealed to present extremely high carrier mobility and rather excellent flexibility regarding adjusting electronic and photocatalytic properties by layers stacking and strain engineering [24,25]. Insulating *h*-BN has a broad bandgap of ~6 eV and no dangling bonds, and it can act as an insulating slab and an excellent substrate [26]. Recent progress reported that two vertical heterostructures, including G/*α*-P and h-BN/*α*-P, inherit the merits of *α*-P, i.e., the direct bandgap and linear dichroism, and further improve the properties of *α*-P concerning easy oxidation when exposed to air [27].

Encouraged by those achievements that were attached to the aforementioned star 2D materials, numerous group IV–VI sheets have been catching people’s interest [28,29,30,31]. Kamal et al. predicted binary isoelectronic counterparts of *α*-P and blue phosphorene (*β*-P), which have almost identical stability in geometric configurations [29]. Among these group IV-VI sheets, several 2D isoelectronic counterparts of *α*-P, such as *α*-SnS [32] and *α*-SnSe monolayers [33], have been manufactured via the exfoliation of their layered bulk whose individual atomic layers are cohered together through weak van der Waals (vdW) force. Indirect-direct transitions that were tuned by stacking coupling for *α*-SnS [30], or by interface engineering for *α*-SnSe [34], which can overcome the electron transition obstacle, are now achieved. Very recently, it was reported that monolayer *α*-CSe, which is an isoelectronic counterpart of *α*-P with direct bandgap, is highly sensitive to ultraviolet-light [31]. To date, most scientific attentions were thrown at *α*-phases of group IV-VI. Fortunately, *β*-P, an allotrope of *α*-P, has been theoretically predicted [35] and experimentally manufactured [36,37], which aroused the investigations concerning *β*-P systems, since their *α*-phases present many novel properties, such as strong in-plane polarization in *α*-SiS [38] and the high thermoelectric figure of merit in *α*-SnSe [39]. Currently, only sporadic *β*-group IV-VI sheets called researches’ attention, such as *β*-SiS [38] and *β*-SnSe [39]. These made us wonder whether another honeycomb covalent network of C and Se atoms naturally exists, how the mechanical and optoelectronic behaviors perform, and whether it is superior to *α*-CSe?

Through the calculations using density functional theory (DFT), we identified a novel ultra-thin stable sheet of carbon selenide (*β*-CSe), which is an isoelectronic counterpart of *β*-P that also has a wide indirect bandgap. The calculations showed that *β*-CSe exhibits slightly anisotropic mechanical characteristics. Its bandgap and band-edges curvature are sensitive to in-plane strain, causing the carrier effective mass to be strain-dependent. A vertical heterostructure (*α*-CSe/*β*-CSe) based on *α*-CSe and *β*-CSe sheets can be constructed at a very small energy penalty, just like the *α*-P/*β*-P heterostructure [40]. Its band alignment belongs to that of a type-II van der Waals *p*-*n* heterojunction. Thus, the electrons and holes are spatially well-separated and distributed in the two layers, respectively. Our results provide a fundamental insight of designing novel structural phase for high-performance photovoltaic devices, and highlight its promising application in photocatalysis.

## 2. Methods

Calculations were performed in the VASP [41,42] code that was based on density functional theory (DFT). The generalized gradient approximations (GGA) [43] that stemmed from Perdew-Burke-Ernzerhof (PBE) [44] as the exchange-correlation potential within the framework of projector augmented wave (PAW) [45] to model the interactions between electrons and ions were used. A vacuum thickness of 20 Å was adopted to inhibit the mendacious interactions between the periodic nanosheets together with plane-wave basis sets of 500 eV energy cutoff. For Brillouin-zone (BZ) integration, Monkhorst-Pack k-meshes with sizes of 31 × 31 × 1 and 5 × 33 × 3 were applied to the sample in the k-space of monolayer *β*-CSe and the bilayer *α*-CSe/*β*-CSe heterostructure, respectively. All of the structures were fully relaxed with a less-than 0.01 eV/Å residual force on each atom. An energy convergence criterion of 10^−6^ eV was set. As the vdW interactions are crucial in predicting the stable heterostructure, a DFT-D2 semiempirical dispersion-correction approach (considering the vdW interactions) was employed in the calculations of the heterostructure [46]. We adopted the Heyd-Scuseria-Ernzerhof-06 (HSE06) [47] hybrid functional to accurately characterize the electronic performance of monolayer and bilayer. The phonon frequencies were garnered by PHONOPY code [48] based on the density functional perturbation theory (DFPT).

## 3. Results and Discussions

### 3.1. Geometric and Electronic Structures

**Figure 1:**
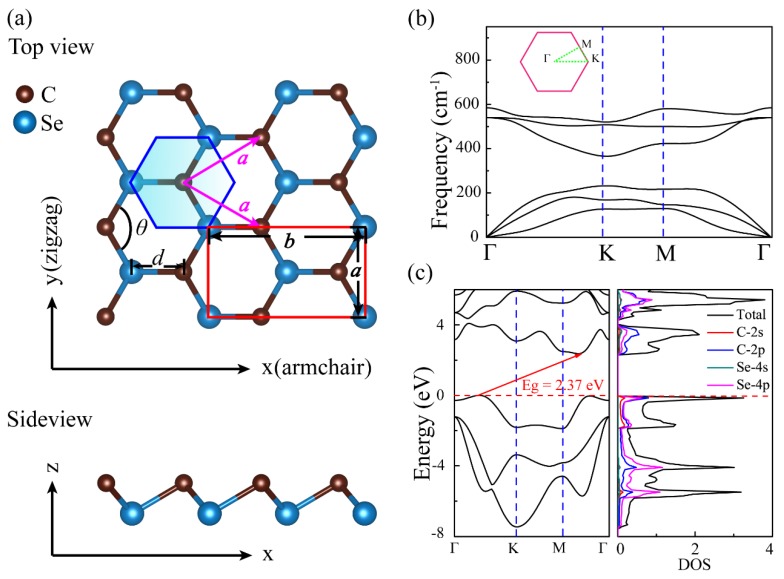
(**a**) The top and side views of monolayer carbon selenide (*β*-CSe). The shade region represents a primitive cell. A rectangular cell in top view also is marked, and used to calculate stress-strain relationships. (**b**) Phonon band diagram, where the panel represents the high-symmetry k-points in the first BZ of the hexagonal reciprocal unit cell. (**c**) Electronic band diagram, and the total and orbital projected partial density of states for the monolayer *β*-CSe, at the HSE06 level. The indirect bandgap of the monolayer is guided by the red arrow and the bandgap value is also provided. The Fermi level is set at zero.

*β*-P, an allotrope of *α*-P with intriguing properties, has been theoretically and experimentally discovered [35,36,37], which is a buckled layer, but it is slightly flat with regard to *α*-P. We substituted for P-P bonds using C-Se bonds in experimentally well-characterized *β*-P skeleton. As such, the buckled atomic configuration, named *β*-CSe, was constructed and is shown in Figure 1a. It is packed into a hexagonal crystal lattice with the *P*3*m*1 space group, one C and one Se atoms are organized in its primitive cell, where each C atom is covalently-linked to its three first-nearest-neighbor Se atoms, and vice versa. These atoms are arranged in the form of a two-atom thick layer with an armchair (zigzag) pattern parallel to *x* (*y*) axis, as shown in Figure 1a. Table 1 lists the well-optimized structural parameters for the *β*-CSe and *β*-P monolayers, which cater to previous results [29,35]. When compared to *β*-P, the different local bonding preference between C and Se atoms alters the buckled height of monolayer *β*-CSe. The buckled angle of *β*-CSe (96.45°) exceeds that of *β*-P (92.907°); therefore, its buckled height is reduced to 1.044 Å with regard to *β*-P (1.238 Å) [35].

The stability of *β*-CSe is first evaluated by the cohesive energy and the phonon calculations. Like the phosphorene allotropes (*α*-P and *β*-P), a cohesive energy of *β*-CSe (−3.79 eV/atom) slightly deviates from *α*-CSe (−3.86 eV/atom). This small difference is comparable to the thermal energy at 300 K [49]. Hence, the monolayer *β*-CSe is predicted to be stable in energy, just as the *α*-CSe sheet. As shown in Figure 1b, no imaginary phonon modes appear in the first BZ, revealing that its structure has outstanding dynamical stability.

Table 2 lists the gaps of *β*-CSe and *β*-P from PBE and HSE06 calculations, which match well with other works [29,35]. Figure 1c shows the electronic structure and total and orbital projected partial densities of states (TDOS and PDOS) of *β*-CSe from HSE06 calculations. PDOS reveals that those states in the valence band maximum (VBM) and the conduction band minimum (CBM), are principally contributed by *p* electrons with less weight of *s* electrons. Specifically, these electronic states approaching the Fermi level have larger contributions from 2*p* electrons of C atoms instead of 4*p* electrons of Se atoms. This is mainly rooted in the fact that C has electronegativity greater than Se [29].

### 3.2. Mechanical Properties

Strain engineering provides an efficient method for investigating the stress-strain relation of 2D sheets [50,51,52]. Specifically, the strain engineering, which is implemented by deliberately imposing mechanical deformation onto an ultrathin sheet, shows that the strain itself potentially serves as a practical tool for investigating the nanostructure response to it. The mechanical behaviors of the monolayer *β*-CSe under uniaxial strain along the armchair ($\varepsilon_{xx}$) and the zigzag directions ($\varepsilon_{yy}$), and the uniform biaxial strain (equiaxial strain $\varepsilon_{xy}$ along *ab* double axes) are calculated. The engineering strain is denoted as $\varepsilon =(n-n_{0})/n_{0}$, where n and $n_{0}$ represent the lattice constants of the strained and equilibrium structures, respectively. The positive *ε* refers to the extension, while negative *ε*, contraction. When imposing the uniaxial strain (the lattice constant in the strain axis is fixed), the in-plane unstrained lattice vector is fully relaxed. For the biaxial strain, only atoms positions in the unit cell are fully relaxed.

**Figure 2:**
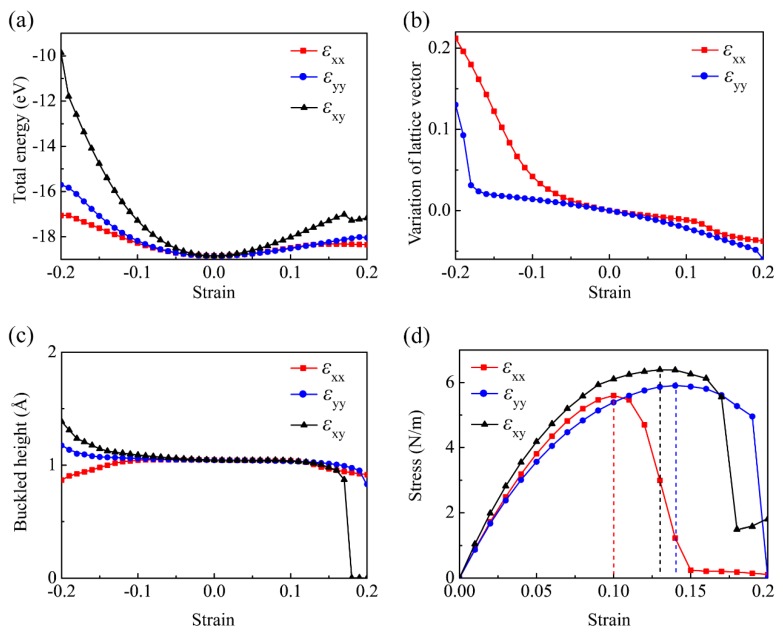
(**a**) Calculated total energy of the monolayer *β*-CSe under strains. (**b**) Variation of the lattice vector perpendicular to the strain direction. (**c**) Buckled height of the monolayer *β*-CSe under the uniaxial and biaxial strains. (**d**) Stress-strain relationships of themonolayer *β*-CSe under three types of strains.

The defined rectangular unit cell (the red frame) in Figure 1a is adopted to conveniently implement the stress-strain simulation. The evolution of its total energy under axial strains is examined first, as shown in Figure 2a. A potential well can be attained, in which the minimum corresponds to the relaxed structure. The curvature of energy under the armchair strain is smaller than that under the zigzag strain, indicating that it is easily deformed along the armchair direction with respect to the zigzag direction. Figure 2b indicates that the monolayer *β*-CSe will contract (expand) in the other perpendicular direction, when it is stretched (compressed) in the armchair- or zigzag-directions. The calculated Poisson’s ratios are 0.14 under the armchair strain and 0.16 under the zigzag strain. A tiny deviation of Poisson’s ratios between the two perpendicular directions indicate the slightly anisotropic nature. The Poisson’s ratios are small, being comparable to those of other 2D materials [49], for example, 0.12 for a monolayer C_3_N, 0.21 for a monolayer BN, and 0.29 for a monolayer SiC, which are all knitted by *sp*^3^ bonds. In order to depict the corrugation of monolayer *β*-CSe, Figure 2c shows the dependences of its buckled height on the uniaxial and biaxial tensions, which is distinct from that of *α*-P [53]. Clearly, Figure 2c shows that the monolayer *β*-CSe expands (compresses) in the *z*-direction when it is contracted (stretched) under all of the strain cases, and thus, negative linear Poisson’s ratio is not appeared like the monolayer *α*-P. The evolutions of the buckled height is different in the three strain cases: the maximum of 1.05 Å appears at the armchair strain of $\varepsilon_{xx}=-0.07$; whereas, the maximum appears at the zigzag strain of $\varepsilon_{yy}=-0.20$; the equibiaxial strain remarkably causes the largest variation of the buckled height in the range of 1.4 Å~0 Å. When the equibiaxial strain exceeds $\varepsilon_{xy}=0.14$, the corrugation rapidly diminishes and vanishes at $\varepsilon_{xy}=0.18$. It implies a structure phase transition from initially the low-buckled configuration to plane structure. Accordingly, orbital hybridization transfers from the weak *sp*^3^ to *sp*^2^ hybridization.

Currently, the interlayer distance of the monolayer *β*-CSe cannot be experimentally determined. Therefore, the in-plane stress (2D stress per unit length) can be used to characterize the strength of the monolayer *β*-CSe [50]. To explore its ideal tensile strength, the uniaxial and biaxial tensile strains are exerted on the relaxed monolayer. As depicted in Figure 2d, the stress-strain curve under the armchair tension almost overlaps that of zigzag tension below a loading of 0.03, i.e., *β*-CSe possesses isotropic in-plane elastic response in this strain interval. As the tension further increases, the stress-strain behavior become nonlinear, and the disparity of elastic response in both orthonormal directions becomes conspicuous. Table 3 summarizes the ideal strengths and critical strains, which reveal the monolayer *β*-CSe can withstand an ultimate stress of 5.6 N/m along the armchair direction, and 5.9 N/m along the zigzag direction. We found that the ideal tensile strengths are nearly identical, just like graphene [54] and silicene [55], which is attributed to the similar rhombic hexagonal structure. Clearly, it can withstand a tensile strain ultimate of 0.1 in the armchair direction and 0.14 along the zigzag direction. These critical strains are found to be small, especially, a critical strain of 0.1 along the armchair direction (as we know, it is the smallest in extensively atomically studied ultra-thin sheets, excluding borophene [50]). Accordingly, *β*-CSe becomes an outstanding candidate for brittle materials. In the case of the biaxial tension, this monolayer can withstand a stress up to 6.4 N/m at $\varepsilon_{xy}=0.13$. Intriguingly, the curve presents an inflexion point with a minimum value under a biaxial strain level of $\varepsilon_{xy}=0.18$, which is responsible for structure phase transition. The Young’s modulus can also be garnered via fitting the initially linear segment of stress-strain curves up to 0.02 for the uniaxial or biaxial strains and Table 3 lists the corresponding values, which reveals that the strength of C-Se bonds may be slightly different between the both orthogonal directions, and it further confirms the anisotropic nature of the monolayer *β*-CSe.

### 3.3. Strain Dependence of the Electronic Structures

**Figure 3:**
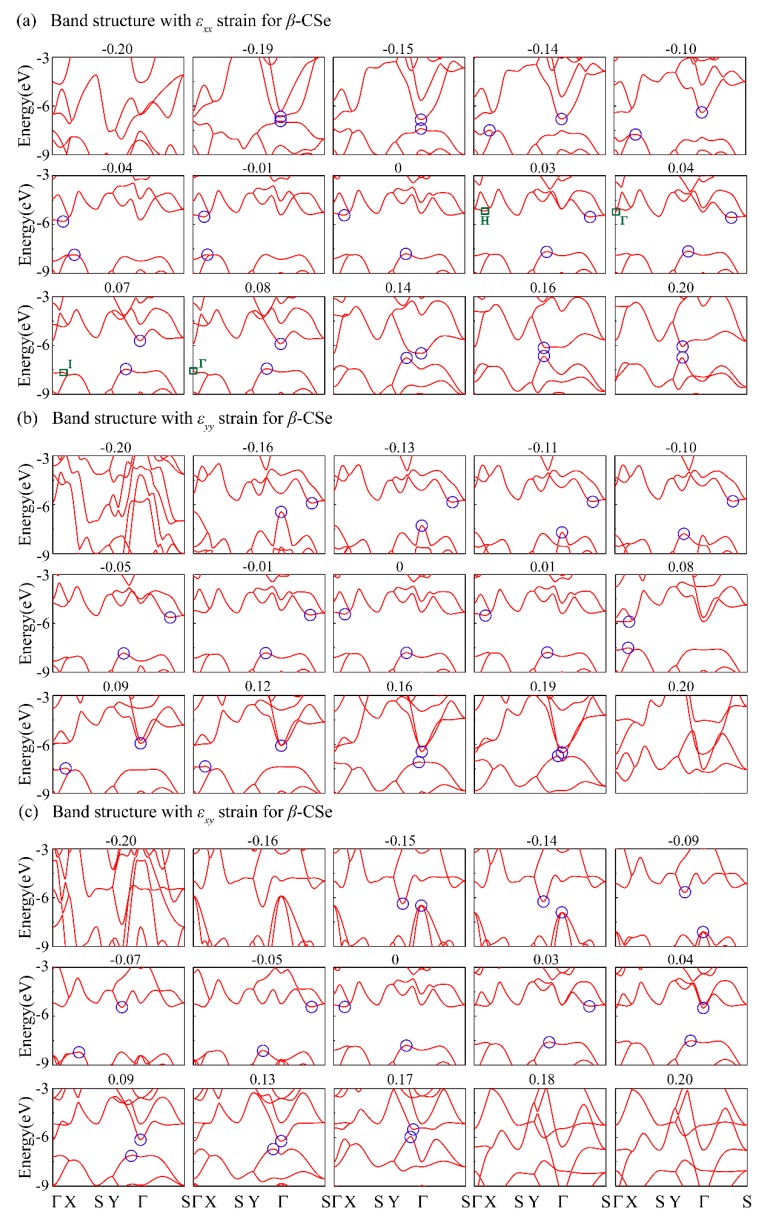
Evaluation of band structures versus armchair strain (**a**), zigzag strain (**b**) and biaxial strain (**c**). The blue circles represent the conduction band minimum (CBM) and valence band maximum (VBM). The energy values are relative to the vacuum level.

Strain engineering has been proved as a commendable avenue for tuning the optoelectronic properties of 2D semiconductors [28,49,56,57]. Consequently, we have carried out calculations regarding the electronic structures of the strained monolayer *β*-CSe. The electronic structures under $\varepsilon_{xx}$ are firstly explored, as shown in Figure 3a. It was found that indirect-to-direct band transition appears under the tension ($\varepsilon_{xx}=0.16$) or compression ($\varepsilon_{xx}=-0.15$) along the armchair direction. The two direct band gaps reside at non-identical high-symmetry points (Γ for tension and the point between Γ and Y for compression). For the case of the band structures under $\varepsilon_{yy}$ or $\varepsilon_{xy}$, as presented in Figure 3b,c, no indirect-to-direct band transition takes place.

Figure 3 illustrates that the positions of VBM and CBM frequently change. The monolayer *β*-CSe at the equilibrium configuration presented indirect behavior with the CBM positioned in Γ-X and VBM lying in Γ-Y, which correspond to the electronic structure of zero strain in Figure 3. Clearly, all of the strained systems experience a semiconductor-metal transition under larger strains, except for large armchair tensile strain. In what follows, specific discussions are focused on semiconductors. For the case of $\varepsilon_{xx}$, with increasing tension, the CBM changes to Γ-S firstly and then shifts to Γ-Y (including Γ), whereas the VBM always lies in Γ-Y. Similarly, with increasing compression, the CBM always lies in Γ-X (including Γ), while the VBM position mainly depends on the competition between the band edges states (Γ-X and X-S). For $\varepsilon_{yy}$, with an increase of tension, the CBM shifts from Γ-X to Γ, while the VBM position is decided by fierce competition between Γ-X and Γ-Y. On the side of compressive strain, the CBM lies in Γ-S and VBM shifts to Γ from Γ-Y with increasing the compression. In the case of $\varepsilon_{xy}$, the CBM position mainly depends on the competition between the band edges states (Γ-Y and Γ-S), while VBM always appears between Γ-Y.

### 3.4. Strain Dependence of the Bandgap

**Figure 4:**
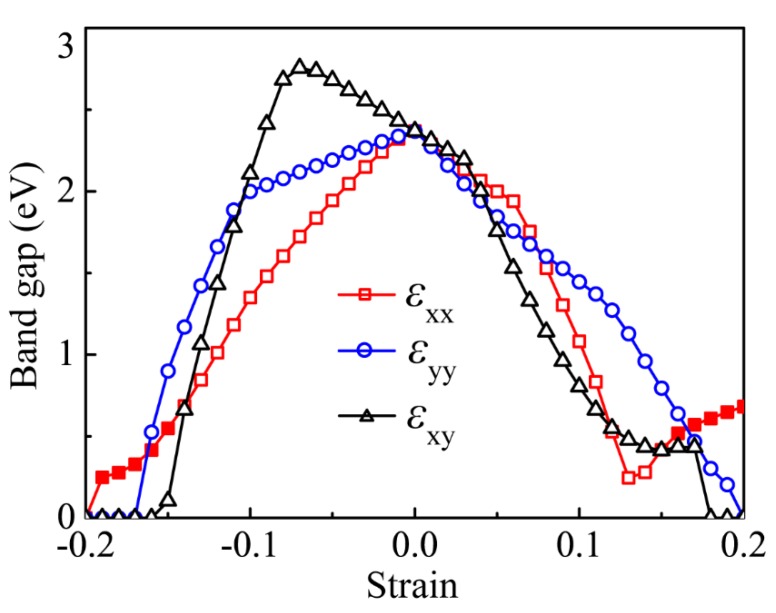
Bandgap of the monolayer *β*-CSe varies with the uniaxial and biaxial strains. Red line, blue line and black line represent the $\varepsilon_{xx}$-, $\varepsilon_{yy}$ -, and $\varepsilon_{xy}$ -induced band gap evolutions, respectively. The hollow and solid symbols indicate the indirect and direct electron transitions, respectively.

The strain-induced electronic structures evolutions issue in significant changes in its band gap. We plot the strain-induced bandgap as functions of $\varepsilon_{xx}$, $\varepsilon_{yy}$, and $\varepsilon_{xy}$, as captured in Figure 4 to clearly elaborate it. One can see that the bandgap is first diminished versus the increasing of the armchair tension until the tensile strain reaches $\varepsilon_{xx}=0.13$, after which the bandgap increases. On the side of contraction, it declines nearly monotonously and even vanishes at $\varepsilon_{xx}=-0.20$. Under $\varepsilon_{yy}$, the band gap narrows to be zero with increasing either the tension or compression. When it was subjected to the equiaxial in-plane tensile strain, the band gap non-monotonously descends and even closes at $\varepsilon_{xy}=0.18$. Upon imposing the compressive strain, the transition location of the band gap from ascension to descension appears at $\varepsilon_{xy}=-0.07$ with increasing the compression, and eventually the bandgap closes. Overall, the armchair-dominant strain issues in an indirect-direct transition despite the indirect characterize being robust. By contrast, the cases are exceedingly distinct for the zigzag and the biaxial strains, where only a transition from semiconductor to metal is observed and the indirect semiconductor nature is well preserved in sizable strain intervals.

### 3.5. The Dependence of Carrier Effective Masse on Strain

**Figure 5:**
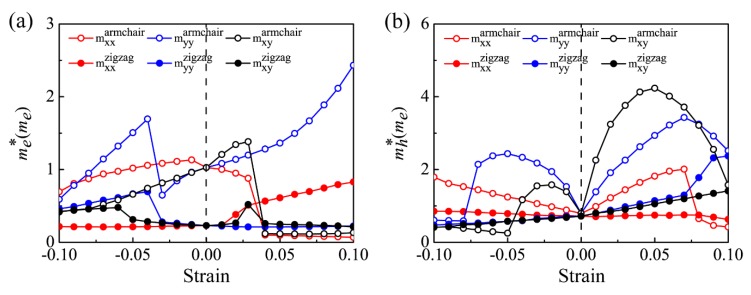
The effective electron (**a**) and hole (**b**) masses as functions of strains ($\varepsilon_{xx}$(red), $\varepsilon_{yy}$ (blue), and $\varepsilon_{xy}$ (black)). Hollow circles and solid circles represent the armchair and zigzag effective masses, respectively. The carrier effective mass is in unit of the static electron mass ($m_{e}$).

The strain in semiconductors can modulate not only the characteristic of electron transitions, band-edges positions, and the band gap, but also the curvature of the electronic band edges. The curvature determines the carrier effective mass $m^{*}:m^{*}=\hbar^{2}{\left(\partial^{2}E/\partial K^{2}\right)}^{-1}$, where *ℏ*, *E*, and *K* refers to the reduced Planck constant, energy, and momentum, respectively. Table 2 summarizes the effective masses of the relaxed *β*-CSe and *β*-P sheets, among which the results of monolayer *β*-CSe have not been reported before and the results of monolayer *β*-P reasonably agree with previous works [58]. Figure 5a,b show the variation of carrier effective mass versus strains in this work. When compared to the zigzag direction, strain exerts a stronger effect on the effective carrier mass of the armchair direction. As for $\varepsilon_{xx}$, the effective electron mass of the armchair direction ($m_{exx}^{armchair}$) presents a sudden drop when the strain exceeds $\varepsilon_{xx}=0.03$, and the situation reappears for the effective hole mass of the armchair direction ($m_{hxx}^{armchair}$) at $\varepsilon_{xx}=0.08$. Specifically, the effective electron mass of the armchair direction far outweighs that of the zigzag direction under $\varepsilon_{xx}<0.04$, which indicates that the preferred transport is along the zigzag direction for electrons in the corresponding strain interval. However, under $\varepsilon_{xx}>0.04$, the armchair direction becomes the dominant direction for electron transport. For the effective hole mass, there is a transition strain ($\varepsilon_{xx}=0.07$), causing a sudden transition from $m_{hxx}^{armchair}>m_{hxx}^{zigzag}$ to $m_{hxx}^{armchair}<m_{hxx}^{zigzag}$ when the $\varepsilon_{xx}$ exceeds the transition strain. It indicates that the prior direction for hole transport has a sudden change under the tensile strain.

$\varepsilon_{yy}$ can also remarkably modulate the effective carrier masses, as shown in the blue lines of Figure 5a,b. For the electrons and holes, a non-monotonous dependence of the effective masses on the zigzag strain was conspicuous. $m_{yy}^{armchair}<m_{yy}^{zigzag}$ remains over the whole strain ranges, where $m_{yy}^{armchair}$ is the carrier effective mass of the armchair direction under the zigzag strain and $m_{yy}^{zigzag}$ is the carrier effective mass of the zigzag direction under the zigzag strain. Consequently, the armchair direction undertakes the dominant transport direction for the electrons and holes, which cannot be changed by the strain. This implies that the transport performances of carriers in the monolayer *β*-CSe preserve mechanical stability.

For the case of $\varepsilon_{xy}$, the biaxial strain provided distinct modulations for the effective carrier mass with regard to the aforementioned uniaxial strain. For electrons, the large anisotropy is decayed and even gradually disappears with increasing compressive biaxial strain, i.e., the favored transport direction has a transition from the single armchair direction to the double directions under $\varepsilon_{xy}=-0.08$. Around $\varepsilon_{xy}=0.04$, a sudden drop of the effective mass along the armchair direction takes place, which causes a preferential transport direction transition from the zigzag direction to the armchair direction. For the holes, at $\varepsilon_{xy}<0$, the anisotropy feature of the effective masses is weakened with an increase of the biaxial compression: at $\varepsilon_{xy}=-0.05$, the effective mass has an apparent transition from $m_{hxy}^{armchair}>m_{hxy}^{zigzag}$ to ($m_{hxy}^{armchair}$ and $m_{hxy}^{zigzag}$ represent the hole effective masses of the armchair and zigzag directions under the biaxial strain, respectively); at $\varepsilon_{xy}=-0.09$, the anisotropy of the effective mass disappears ($m_{hxy}^{armchair}=m_{hxy}^{zigzag}$); at $\varepsilon_{xy}>0$, the holes are always heavier than the electrons. Clearly, the larger anisotropy in the carrier effective mass during load will result in anisotropic carrier mobility, and will give further rise to direction-dependent conductivity. Overall, the sudden and frequent shift about the effective masses of the electrons and the holes leads to competition between two orthonormal directions concerning the preferred transport.

The effective mass shown in Figure 5 is directly related to the electronic structures shown in Figure 3. Particularly, the dramatic shift regarding the effective mass is due to the fierce competition of band-edge extremes. In order to have an in-depth understanding about the dramatic shift, only the armchair strain is taken as a typical reference since the sharp change of effective carrier mass in other strain cases is similar in physical mechanism. Specifically, the dramatically downward change of the effective electron mass in the armchair direction around $\varepsilon_{xx}=0.04$ in Figure 5a, consults the band structure of Figure 3a along Γ-X (armchair). When compared to the state H in Figure 3a at $\varepsilon_{xx}=0.03$, the downward shift of state Γ under $\varepsilon_{xx}=0.04$ obviously strengthens the band dispersion along the armchair direction, and it thus dramatically decreases the effective electron mass at $\varepsilon_{xx}=0.03$. The armchair CBM is departed from H to Γ when the strain exceeds $\varepsilon_{xx}=0.03$. Thereby, the calculated effective electron mass from state Γ is smaller, because of strengthened dispersive at the new armchair CBM. Another dramatic downshift in the effective hole mass appears at $\varepsilon_{xx}=0.08$ in Figure 5b, which is closely related to the band structure of Figure 3a at $\varepsilon_{xx}=0.08$. Here, the energy of the valence-band state Γ surpasses the state I and then becomes the new armchair VBM. The effective hole mass of armchair direction is now calculated according to this emerging armchair VBM (the state Γ) rather than the state I.

### 3.6. The Type-II vdW p-n α-CSe/β-CSe Hetrostructure as a Metal-Free Photocatalyst

**Figure 6:**
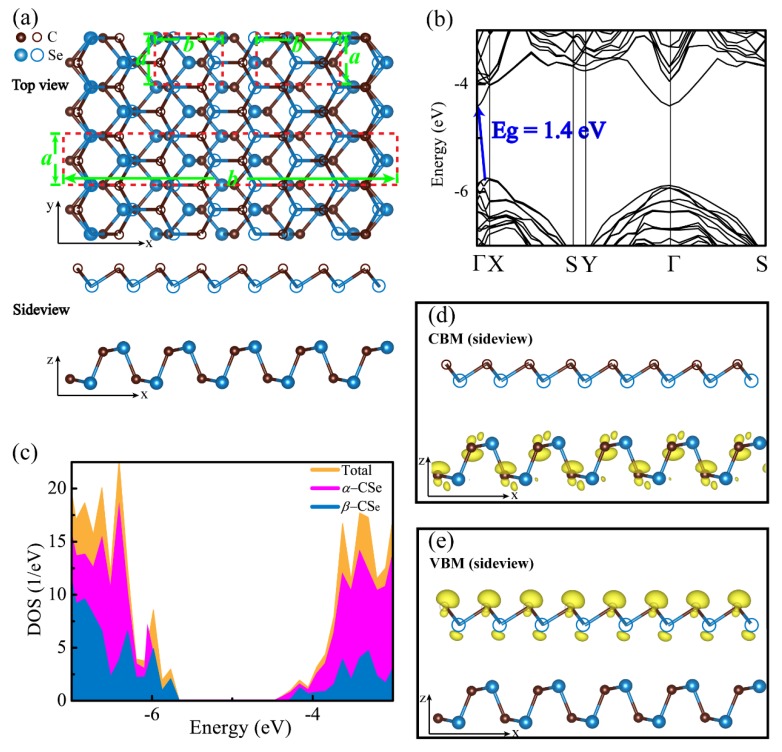
Top- and side-views (**a**) for the *α*-CSe/*β*-CSe vdW hetrostructure. The three red dashed frames are the rectangle unit cell of *α*-CSe, *β*-CSe and the heterostructure, respectively. The band structure (**b**) and density of states (**c**) refer to vacuum level. The band decomposed charge density of the CBM (**d**) and VBM (**e**) in the *α*-CSe/*β*-CSe vdW heterostructure. The value of isosurfaces is 0.03 e/Å^3^. The hollow and solid spheres represent the top and bottom layers, respectively.

As a new degree of freedom, which introduces interfacial coupling, is also expected to tailor optoelectronic performance. This stimulates us to propose the *α*-CSe/*β*-CSe vdW heterostructure. The unit cell of the proposed vdW nanocomposite was constructed by placing the super cell, which includes 4 × 1 rectangle unit cells of *β*-CSe on the top of the super cell includes 5 × 1 unit cells of *α*-CSe. The fully-optimized atomic motif that was obtained from the calculations of DFT+D2 function is shown in the upper (topview) and lower (sideview) panels of Figure 6a. The well-optimized lattice parameters of 5 × 1 super cell of *α*-CSe and 4 × 1 rectangle super cell of *β*-CSe are *a* = 3.048 Å and *b* = 21.47 Å, and *a* = 3.057 Å and b = 20.89 Å, respectively. Such a complex has the well-optimized lattice constants of a = 3.051 Å and *b* = 21.08 Å. Consequently, the overall induced largest mismatch is 1.82% in α-CSe along the *b*-direction, allowing for one to engineer its optoelectronic properties at a low energy penalty.

The effect of vdW interaction can be authenticated via analyzing the electronic structure. As shown in Figure 6b, the hybrid *α*-CSe/*β*-CSe vdW heterojunction has an indirect band gap of 1.4 eV at the HSE06 level, and the VBM is positioned at a non-high-symmetry k-point along Γ-X, whereas the CBM appears at Γ. Obviously, the vdW interaction does reduce the band gap and it reshapes energy band extremum (EBE). In order to uncover the origin of EBE, DOS is plotted, as presented in Figure 6c. The sulfur yellow and magenta (blue areas) in Figure 6c represent the contributions from the relaxed heterostructure and the pure *α*-CSe (*β*-CSe) layer, respectively. The results indicate that the CBM are dominated by *α*-CSe, whereas the VBM is mainly rooted in *β*-CSe, i.e., the vdW interaction in this hybrid heterojunction changes the positions of EBE in comparison with the free-standing monolayer *β*-CSe, and forms an atomically sharp type-II vdW heterostructure. In this type-II heterostructure, the photon-generated electron-holes pairs should be separated in space, in the form of electrons and holes that were allocated in different layers. In-depth analysis concerning the partial charge densities of CBM and VBM further supports that this heterostructure belongs to Type-II vdW heterostructure, because the CBM (Figure 6d) and VBM (Figure 6e) are mainly originated from the states of the *α*-CSe layer and the *β*-CSe layer, respectively. These separated optically active states in space are equivalent to spontaneously separated carriers that were generated from photon, which helps to improve the solar energy conversion efficiency. The band alignment of *α*-CSe/*β*-CSe also supports this conclusion, as follows. To achieve the band alignment between *α*-CSe and *β*-CSe, we firstly ascertain the positions of band edges with respect to the vacuum level. The positions of band edges (referred to the vacuum level) in the *α*-CSe and *β*-CSe sheets before and after contacting can be obtained by solving the Kohn–Sham equation. More specifically, after *α*-CSe contacts *β*-CSe, the bandgap (1.52 eV) of the *α*-CSe layer is approximately unchanged when compared with that of the isolated one [31], spanning an energy range from −5.86 to −4.34 eV, while the band gap of the *β*-CSe monolayer apparently reduces to 2.17 eV with regard to that of the isolated monolayer *β*-CSe, spanning from −5.74 eV to −3.57 eV. Therefore, the CBM and VBM come from different layers, resulting in a typical type-II vdW heterostructure.

**Figure 7:**
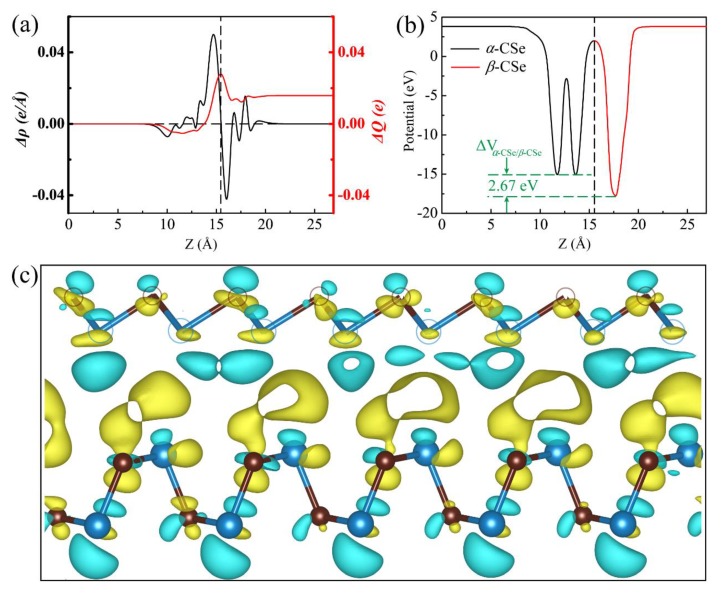
(**a**) The planar-averaged differential charge density Δρ(z) of the *α*-CSe/*β*-CSe vdW hetrostructure (black) and the amount of transferred charge ΔQ(z) as a function of position along the *z* direction (red). (**b**) *xy*-averaged electrostatic potential shape through the interface of the *α*-CSe/*β*-CSe vdW hetrostructure. (**c**) The sideview of the charge density difference for the *α*-CSe/*β*-CSe vdW hetrostructure. The value of isosurfaces is 0.0004 e/Å^3^. The yellow and cyan areas exhibit the accumulation and depletion of charges, respectively.

In order to precisely characterize such vdW coupling between the *α*-CSe layer and the *β*-CSe slab, we calculated the planar-averaged charge density difference (CDD), which is defined as $\Delta \rho (z)=\rho_{\alpha -CSe/\beta -CSe}-\rho_{\alpha -CSe}-\rho_{\beta -CSe}$, here $\rho_{\alpha -CSe/\beta -CSe}$,$\rho_{\alpha -CSe}$, and $\rho_{\beta -CSe}$ represent the charge densities of the hybrid heterojunction architecture, the pure *α*-CSe slab, and the pure *β*-CSe sheet, respectively. Figure 7a shows the planar-averaged CDD (black line) of the *α*-CSe/*β*-CSe heterostructure as a function of the *z*-axial position. The change of position-dependent Δρ(z) at interface evidences that the *β*-CSe layer contributes electrons to the *α*-CSe layer, which induces a slight *n*-type doping in the *α*-CSe layer and a *p*-type doping in the *β*-CSe layer. To quantify the transfer of charge, the electron-transfer quantity (red line) up to *z* point can be acquired by $\Delta Q(z)=\int_{-\infty }^{z}\Delta \rho (z^{\prime })dz^{\prime }$. The electron gain in the *α*-CSe layer is 0.028 *e*, as calculated from the value of ΔQ(z) at the charge-transfer complex interface. Such small charge transfer reveals a weak interlayer coupling between the *α*-CSe layer and the *β*-CSe layer. To further unveil the charge-transfer mechanism, Figure 7c provides the three-dimensional isosurface of the CDD, where the charge accumulation (yellow) and the depletion (cyan) of electrons across the interface are intuitively illustrated. Apparently, the charge density redistributes in the interface region of the heterostructure. The holes accumulate near the *β*-CSe region, whereas the electrons accumulate in the region near the *α*-CSe layer. In the formation of a *p*-*n* α-CSe/β-CSe vdW heterostructure, charge density redistributes and the Fermi level is driven to the CBM of α-CSe and VBM of *β*-CSe after they contact. Meanwhile, the *α*-CSe layer acts as a role of the electron acceptor, while the *β*-CSe layer served as an electron donor.

Figure 7b shows the plane-averaged electrostatic potential along the direction normal to the surface of the heterostructure. The potential drop ($\Delta V_{\alpha -CSe/\beta -CSe}$) across the bilayer is found to be 2.67 eV. Such a potential difference indicates an appreciable built-in electronic field across the interface, which may be ascribed to the charge transferred. Meanwhile, the carrier transport is inevitably influenced, i.e., the excitonic behaviors of the *α*-CSe/*β*-CSe vdW heterostructure is fairly different from that of the isolated CSe monolayers, because the gradient of the potential across the interface confines the electrons and holes within the different sheets. Bader charge analysis can further support the formation of built-in electronic field. There is about 0.027 *e* transferring from the *β*-CSe layer to the *α*-CSe slab, which is qualitatively consistent with the aforementioned value of ΔQ(z) at the *α*-CSe/*β*-CSe interface. This phenomenon may signify that an apparent space-charge region is formed in this interface. Net positive and negative charges are gathered in different layers, which induce a polarized built-in electric field that was directed from *β*-CSe to *α*-CSe. The polarized built-in electric field across the interface imposes another force on carriers (holes and electrons), which is opposite with the diffusion force. The balance between electric field force and diffusion force are propitious to inhibit the recombination of the electrons and holes.

**Figure 8:**
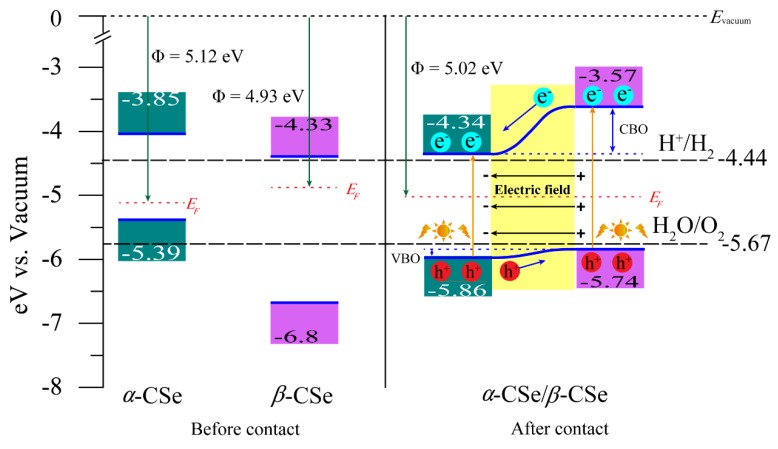
Diagram of the band alignments before and after the isolated monolayer *α*-CSe and *β*-CSe contact. The work functions (Φ) for the free-standing monolayer *α*-CSe, *β*-CSe and bilayer *α*-CSe/*β*-CSe are also provided. The vacuum level $E_{vacuum}$ is set to 0 eV and $E_{F}$ denotes the Fermi level.

Having inerrably confirmed the type-II heterostructure and clearly understanding a full picture of its charge transfer, it is now imperative to investigate its redox power, as this is responsible for its photocatalytic performance. Erenow, which is the redox power in the isolated sheets, is firstly evaluated. Figure 8 indicates that the band edge alignments before and after contacting and the water redox potentials. The water redox potentials are constant, coming from the experimental measurement [30]. Before contacting, as for *α*-CSe, the potential of the CBM is positioned at 0.59 eV above the reduction potential of $H^{+}/H_{2}$(−4.44 eV), enabling the generation of *H*_2_, while the oxidation potential of *H*_2_*O*/*O*_2_ (−5.67 eV) lies below its VBM, indicating that *O*_2_ cannot be spontaneously achieved. The contrary situation takes place in *β*-CSe, i.e., *β*-CSe hardly generates *H*_2_ spontaneously, since its CBM is close to the reduction potential of $H^{+}/H_{2}$, while it possesses prominent oxygen evolution ability, since its VBM is largely lower than the oxidation potential of *H*_2_*O*/*O*_2_. Overall, both the isolated *α*-CSe and *β*-CSe monolayers are not suitable as an intrinsic photocatalyst.

After contacting, the interface charge-transfer drives the Fermi level to move, which brings the movement of band-edge positions of the two CSe sheets. Thus, the band-edges bestride the water redox potentials and make the hybrid *α*-CSe/*β*-CSe heterostructure an excellent candidate for applications in sunlight-driven photocatalysis. It is easy to understand the underlying mechanism by the calculated work functions of the free-standing *α*-CSe and *β*-CSe monolayers. The work function is defined as $\Phi =E_{vacuum}-E_{F}$, where, $E_{vacuum}$ and $E_{F}$ represent the vacuum energy level and Fermi level, respectively. Figure 8 marks the work functions of the free-standing monolayers. A large difference of work function between *α*-CSe (5.12 eV) and *β*-CSe (4.93 eV) signifies that the electrons in the *β*-CSe monolayer will spontaneously transfer to the *α*-CSe monolayer once they contact each other until the $E_{F}$ of the two monolayers are aligned. This will result in the heterojunction possessing a nearly middle work function of 5.02 eV in comparison with that of the isolated monolayers. This result matches well with the aforementioned CDD, ΔQ(z), and Bader charge. Typical type-II band alignment feature also directly resulted in conduction band offset (CBO) and valence band offset (VBO) on both sides of the interface, which is a vital factor in determining the photocatalytic ability $\Delta_{VBO}$ and $\Delta_{CBO}$, 0.07 eV and 0.77 eV, enable the photo-generated carriers to participate in the $H^{+}$ reduction reaction (hydrogen evolution) and the $OH^{-}$ oxidation reaction (oxygen evolution).

Actually, as for the water redox reactions, the process has close connection with the built-in interface electric field and the band offset (BO). Under solar light irradiation, the electrons in this heterostructure are excited, where the electrons in valence bands (VBs) thus transfer to conduction bands (CBs), and holes simultaneously leave in their VBs. The built-in electric field across the interface and its BO give rise to the band edges bending. In general, the upward band bending facilitates the holes migrating upward, and it impedes the electrons from moving. Conversely, electrons can transfer downward along the band bending, while holes are not allowed to move freely [59]. More specifically, CBO facilitates the photo-generated electrons to transfer from the CBs of the *β*-CSe layer to the CBs of the α-CSe layer. On the contrary, VBO promotes the holes to move from the VBs of the *α*-CSe layer to the VBs of the *β*-CSe layer. In the meantime, the balance between built-in electronic field and BO prevents the opposite movements of photo-excited carriers. Consequently, under the combined effect of BO and the built-in electric field, those photo-excited carriers are effectively separated and confined in the different layers. Such a separation in space represses the recombination of electron-hole pairs and effectively prolongates their lifetime, which contributes to enhancing the photocatalytic efficiency of the heterojunction. In more detail, massive reductive electrons staying in the CBs of the *α*-CSe sheet are capable of driving the hydrogen evolution reaction, and simultaneously $H_{2}O/OH^{-}$ can also be oxidized to $O_{2}$ by substantial oxidizing holes that are located in the VBs of the *β*-CSe layer. Overall, the *α*-CSe sheet coupled with the *β*-CSe monolayer, as a metal-free photocatalyst, enables a higher sunlight-harvest efficiency for photocatalytic water splitting in comparison with the intrinsic unsuitable photocatalytic CSe sheets. Experiments are expected to further corroborate our theoretical findings that were reported in this work [60].

## 4. Conclusions

In summary, we predicted a stable blue-phosphorus-like monolayer *β*-CSe that is based on DFT. *β*-CSe sheet exhibits slightly anisotropic mechanical characteristics: it can endure an ultimate stress of 5.6 N/m at $\varepsilon_{xx}=0.1$ along the armchair direction, and 5.9 N/m at $\varepsilon_{yy}=0.14$ along the zigzag direction. As an isoelectronic counterpart of blue phosphorene, *β*-CSe also presents a wide indirect bandgap (2.37 eV) that is sensitive to the in-plane strain. Thus, the carrier effective mass is strain-dependent. Therefore, a strain-sensitively transport direction displays in *β*-CSe. The indirect character of band gap is robust in *β*-CSe, except that armchair-dominant strain can drive an indirect-direct transition. We propose a heterojunction built by the *β*-CSe sheet covering the *α*-CSe sheet (*α*-CSe/*β*-CSe). The band alignment demonstrates that the *α*-CSe/*β*-CSe interface is a type-II van der Waals *p*-*n* heterojunction. An appreciable built-in electric field across interface, which is caused by the charges transferring from *β*-CSe layer to *α*-CSe layer, renders energy bands bending, and causing photo-generated carriers to be spatially well separated. Therefore, *α*-CSe/*β*-CSe heterojunction, as a metal-free photocatalyst, is endued an enhanced solar-driven redox ability for photocatalytic water splitting by lessening the electron-hole-pair recombination. This study provides a fundamental insight of designing novel structural phase for high-performance light-emitting devices, and bodes well for application to photocatalysis.

## Figures and Tables

**Table 1:** The cohesive energy and optimized structural parameters of the monolayer *β*-CSe and blue phosphorene (*β*-P).

Structure	Space Group	Cohesive Energy (eV/atom)	Lattice Constants (Å)		Bond Length (Å)	Bond Angle (deg)
			*a*	*b*		
*β*-CSe	*P*3*m*1	−3.79	3.065	5.22	2.055	*θ* = 96.45
*β*-P	*P*3*m*1	−5.23	3.28/		2.261	*θ* = 92.907

**Table 2:** The bandgaps and carrier effective mass of monolayer *β*-CSe and *β*-P. Indirect bandgap is marked as In in parenthesis.

Material	PBE Gap (type) eV	HSE Gap (type) eV	m^*^_h_/*m*_e_ Zigzag Direction	m^*^_h_/*m*_e_ Armchair Direction	m^*^_e_/*m*_e_ Zigzag Direction	m^*^_e_/*m*_e_ Armchair Direction
*β*-CSe	1.54 (In)	2.37 (In)	0.718	0.795	0.23	1.027
*β*-P	1.94 (In)	2.7 (In)	0.588	0.486	0.353	0.794

**Table 3:** The ideal strengths (*f*), critical strains ($\varepsilon_{c}$), Young’s modulus and Poisson’s ratios of monolayer *β*-CSe under the three strain styles.

Direction	*f* (N/m)	$\varepsilon_{c}$	Young’s Modulus (N/m)	Poisson’s Ratio
Armchair	5.6	0.10	86.14	0.14
Zigzag	5.90	0.14	83.47	0.16
Biaxial	6.4	0.13	99.15	0.09
